# Structure, Optical and Electrical Properties of Nb(Zn) Doped Sol–Gel ITO Films: Effect of Substrates and Dopants

**DOI:** 10.3390/molecules29225480

**Published:** 2024-11-20

**Authors:** Mariuca Gartner, Anna Szekeres, Simeon Simeonov, Maria Covei, Mihai Anastasescu, Silviu Preda, Jose Maria Calderon-Moreno, Luminita Predoana, Hermine Stroescu, Daiana Mitrea, Madalina Nicolescu

**Affiliations:** 1 Institute of Physical Chemistry “Ilie Murgulescu”, Romanian Academy, 202 Splaiul Independentei, 060021 Bucharest, Romania; mgartner@icf.ro (M.G.); manastasescu@icf.ro (M.A.); predas01@yahoo.co.uk (S.P.); lpredoana@yahoo.com (L.P.); dmitrea@icf.ro (D.M.); mnicolescu@icf.ro (M.N.); 2 Institute of Solid State Physics, Bulgarian Academy of Sciences, 72 Tsarigradsko Chaussee, 1784 Sofia, Bulgaria; simeon@issp.bas.bg; 3 Department of Product Design, Mechatronics and Environment, Transilvania University of Brasov, 29 Eroilor Bd., 500036 Brasov, Romania; maria.covei@unitbv.ro

**Keywords:** sol–gel films, Nb(Zn) doped ITO thin films, microstructure, optical properties, electrical properties

## Abstract

We present comparative studies of sol–gel ITO multilayered films undoped and doped with Nb or Zn (4 at.%). The films were obtained by successive depositions of five layers using the dip-coating sol–gel method on microscopic glass, SiO_2_/glass, and Si substrates. The influence of the type of substrates and dopant atoms on the structure and optical properties of the sol–gel ITO thin films is examined and discussed in detail. XRD patterns of these layers showed a polycrystalline structure with an average crystallite size of <11 nm. Raman spectroscopy confirmed the chemical bonding of dopants with oxygen and showed the absence of crystallized Nb(Zn)-oxide particles, indicated by the XRD pattern. Spectroscopic Ellipsometry and AFM imaging revealed a clear dependence of the optical parameters and surface morphology of the ITO and ITO:Nb(Zn) thin films on the type of substrates and dopants. The analysis of the current-voltage and capacitance-voltage characteristics of the Al/ITO/Si structures revealed the presence of charge carrier traps in the ITO bulk and the ITO-Si interface. The densities of these traps are obtained and the character of the current transport mechanism is established.

## 1. Introduction

Why Indium tin oxide (ITO)? Although Indium (In) has become scarce and there is a trend to replace it with more accessible natural elements, it is still the most suitable material for a lot of applications and, therefore, still intensively used. Some of the advantages of ITO are as follows:

- It is simultaneously electrically conductive (a low electrical resistivity of ~10^−4^ Ω·cm) and optically transparent (more than 80%) [1];
- It is durable and can withstand various environmental conditions, which is essential for the longevity of electronic devices;
- ITO is a ternary composition of indium, tin, and oxygen in varying proportions. By varying the ratio of the elements, the transparency of ITO can be adjusted. As a thin film, ITO can have an optical transmittance well above 80%.

These characteristics make ITO a superior choice over other materials, such as zinc oxide or silver nanowires, which may not offer the same level of performance. Besides all these advantages, ITO also presents some disadvantages as listed below:

- The scarcity and high cost of indium can increase the manufacturing costs of devices using ITO;
- ITO can be brittle, leading to potential durability issues in flexible electronics applications. For this reason, other materials are being researched nowadays, such as carbon nanotubes, graphene, and conductive polymers that could provide similar benefits without the associated drawbacks.

The properties of thin ITO films are strongly affected by the conditions of the obtaining methods, each presenting a series of advantages and disadvantages. Furthermore, post-deposition annealing has a strong impact on the film structure [2,3,4,5] which can also be influenced by the type of substrate [3,4]. For specific applications, different substrates are used; for example, glass or silica substrates are suitable for transparent conductive electrodes and biosensors [6,7] and gas sensors [3,8,9] while Si substrates are used in photovoltaics, solar cells, etc. [10,11,12]. Regarding the substrate type, there are also reports concerning the use of quartz, alumina, or polymer substrates (PDMS, PET, and PC for the development of flexible devices) [4,13,14,15,16].

Besides the usual applications listed above (optoelectronic industry [10,15,16] or sensors [3,8,9]), ITO coatings can operate at temperatures up to 1400 °C and can be used in harsh environments, such as gas turbines, jet engines, and rocket engines [17].

To improve the properties of ITO layers, they have been doped (see Table 1) and the influence of dopants on their properties has been studied according to the application.

Although in Table 1 we considered the recent developments in ITO-doped films in the last five years, there are the latest publications related to the preparation and study of pure ITO films [24,25,26]. All this shows a continuing interest in the preparation of pure and doped ITO layers in various fields of applications.

In our previous research [3,22,27], we have explored the structural, morphological, and optical properties of ITO thin multilayered films prepared by the sol–gel method. We have extended our research to multilayered films doped with metal impurities aiming to fabricate multilayered sol–gel ITO films with enhanced TCO properties. These studies have demonstrated the possibility of obtaining competitive TCO films based on ITO by the sol–gel method. To improve the TCO properties of the ITO sol–gel films, we have doped them with Nb and Zn and we have studied the influence of the dopants on the structure and optical properties [3,22].

In the present study, based on our previous experience, we continue the investigation of the effects of the dopant (Nb or Zn) on the properties of sol–gel ITO thin films deposited on glass, SiO_2_/glass, and Si substrates. These substrates were selected with a view to their further possible applications. Based on our experience, we have selected five-layered sol–gel dip-coated thin ITO films, which are still very thin and have a stable structure. The influence of the type of substrates and dopant atoms on the structure, morphology, and optical properties of these ITO films is examined and discussed in detail. The electrical properties of the sol–gel films are investigated on ITO:Nb(Zn) deposited on Si substrates. The analysis of the current-voltage (*I-V*) and capacitance-voltage (*C-V*) characteristics of the Al/ITO/Si structures reveals the presence of charge carrier traps (deep levels) in the ITO bulk. The concentration and energetic parameters of these deep levels are obtained and the character of the current transport mechanism is established.

## 2. Results and Discussion

### 2.1. Structural Characterization by XRD

**Figure 1:**
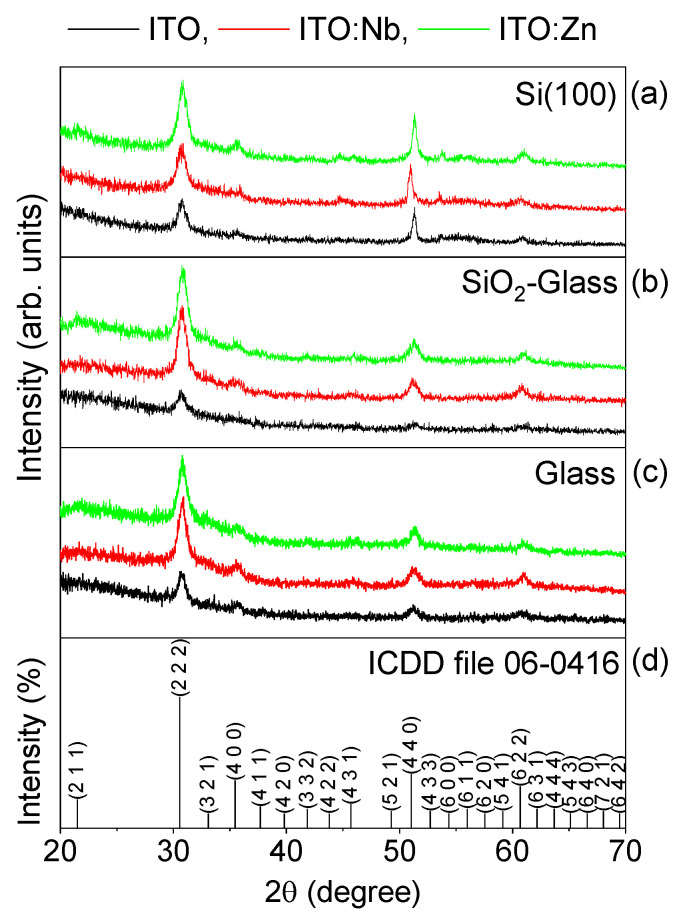
XRD patterns of the undoped (black line), Nb- (red line), and Zn-doped ITO (green line) thin films, deposited on Si(100) (**a**), SiO_2_/glass (**b**), glass (**c**) substrates. The ICDD file no. 6-0416 is given as a reference in (**d**).

Figure 1 shows the XRD diffractograms of the undoped, Zn- and Nb-doped ITO thin films deposited on the three substrate types. All thin films are polycrystalline, with diffraction lines matching well against ICDD file no. 6-0416, corresponding to the cubic bixbyite-type In_2_O_3_ structure. None of the patterns exhibits diffraction lines of tin oxide phases, indicating that the Sn ions substitute for In ions within the indium oxide lattice, thereby maintaining the In_2_O_3_ structure. Also, no diffraction lines from other phases are observed, indicating the incorporation of the Zn and Nb dopants into the Sn-doped In_2_O_3_ lattice.

In Figure 1, only weak variations in the Bragg reflection position and shape with the type of substrates and doping atoms can be noticed. Generally, the XRD patterns of ITO films deposited on Si substrate show more intense and slightly narrower diffraction lines, indicating the formation of larger-sized crystallites when the substrate has a crystalline structure.

Table 2 lists the lattice parameter and crystallite size only for the crystal plane with Miller indices (222). It can be noticed that the interplanar spacing for the Zn-doped ITO samples is the smallest, for each type of substrate. In octahedral coordination, the cation radius of Zn^2+^ (0.74 Å) is smaller than the radius of In^3+^ (0.8 Å). This reduction in interplanar spacing can be attributed to the substitution of more In^3+^ ions with Zn^2+^ ions, as well as the replacement of In^3+^ ions with Sn^4+^ ions. Conversely, the interplanar spacing of the undoped ITO and Nb-doped ITO samples is similar, even though the cation radius of Nb^5+^ (0.64 Å) in octahedral coordination is smaller than both cation radii of In^3+^ and Sn^4+^. This behavior can be explained by Nb^5+^ entering the crystal lattice more likely at interstitial sites rather than substituting for In^3+^.

For the doped ITO films broader and more intense reflections are observed, indicating that the presence of dopants might generate a larger amount of nucleation centers, but the nanocrystallites grow to smaller sizes (Table 2).

**Figure 2:**
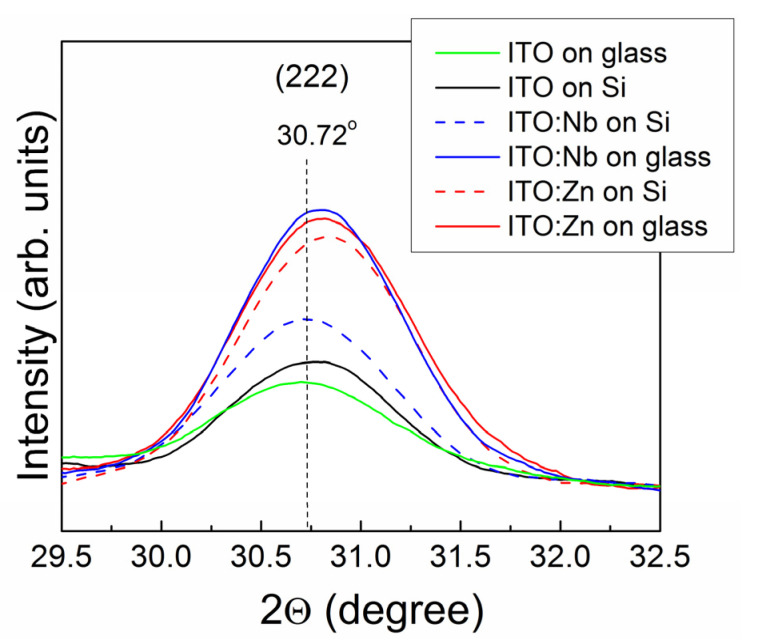
The deconvolution of the main (222) reflections into Gaussian profiles as a function of the substrate type. The diffractograms are corrected with the baselines.

Both doping atoms promote the crystallization process, and the evolution is highlighted in Figure 2, where the changes in the Gaussian profiles of the main (222) Bragg reflections become more apparent. For the undoped ITO films, the type of substrate has a weak influence on the crystallite sizes and ITO lattice itself as the position and shape of the corresponding (222) reflections are similar. In contrast, for the Nb(Zn) doped ITO films, depending on the type of substrate, both the position and shape of the reflections change, visually displaying the results given in Table 2.

### 2.2. Morphological Characterization by AFM Imaging

**Figure 3:**
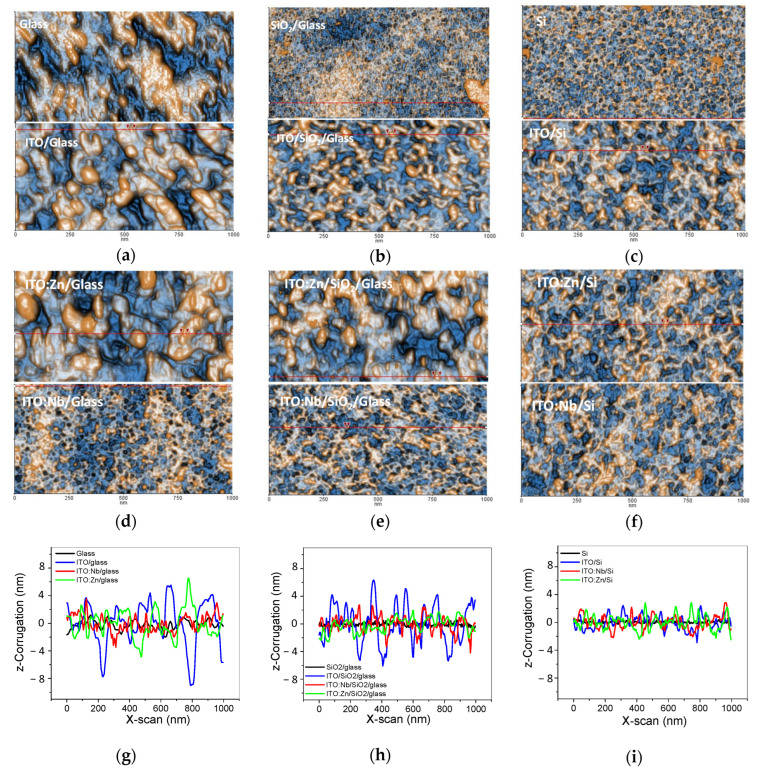
2D AFM images of bare substrates/undoped ITO (**a**–**c**), Zn-doped/Nb-doped ITO films (**d**–**f**); superimposed characteristic line-scans of the undoped and Zn(Nb) doped ITO films on glass (**g**), SiO_2_/glass (**h**) and Si (**i**) substrates. The line-scans were collected at the positions indicated in the AFM figures by horizontal red lines; random surface particles are marked along the lines between two red small arrows.

Before deposition of ITO films, the surface of the substrates was scanned by AFM, presented in the upper half images in Figure 3a–c. The images show that the Si wafer and SiO_2_/glass substrates are very smooth, with RMS roughness values around 0.2 nm, while the glass substrate is slightly rougher (1.2 nm). The AFM images of the undoped ITO films (bottom half images in Figure 3a–c) reveal the clear influence of the substrate on the morphology of the indium tin oxide films: smaller particles are formed on the Si substrate (Figure 3c), with slightly bigger ones on SiO_2_/glass (Figure 3b) and much bigger on the glass substrate (Figure 3a). This can be correlated with the thickness of the films (Section 2.4.2.) and with their degree of crystallinity (Figure 2).

The ITO films formed on Si (RMS = 1.06 nm) and glass (RMS = 3.61 nm) substrates are relatively thin and therefore their topography is affected by the crystallinity and surface roughness of the substrate [28]. On the other hand, in the case of doping, the Zn-doped ITO films are similar to the undoped ones regarding their morphological characteristics. This may be due to the fact that the ITO:Zn films are thinner on the glass than those of ITO:Nb and therefore the influence of the glass surface morphology is stronger for them. For the Nb-doped ITO films, the substrate “fingerprint” is maintained only for the film deposited on Si (Figure 3f), while in the case of glass (Figure 3d) and SiO_2_/glass (Figure 3e) the morphology consists of small particles and small pores.

**Figure 4:**
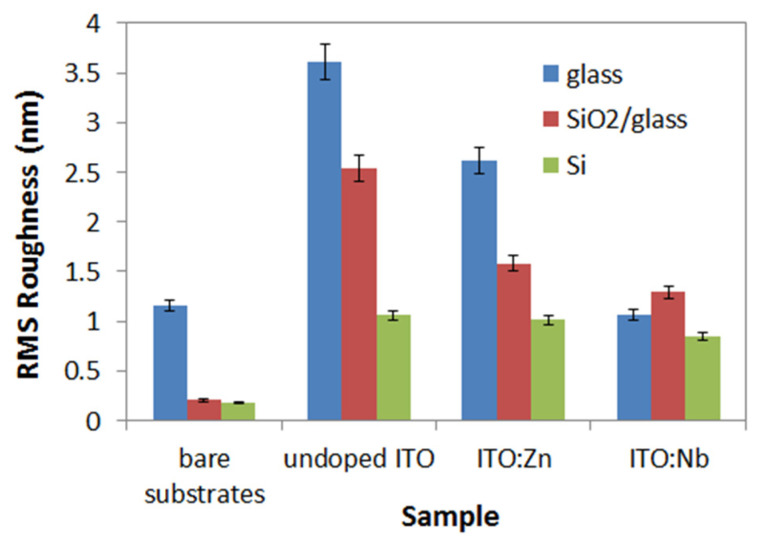
RMS roughness of undoped and Zn(Nb) doped ITO films in comparison to the RMS values of the bare substrates.

The topographic main features are well expressed in Figure 3g–i, where the surface corrugation presented in the form of line-scan decreases in the sequence: glass (Figure 3g); SiO_2_/glass (Figure 3h) and Si (Figure 3i). A similar behavior can be observed in Figure 4, where the RMS roughness values are plotted for all ITO films as a function of the substrate. In each series of films, the smoothness is increased from glass to Si, and from undoped to Zn- and Nb-doped.

### 2.3. Morphological Characterization by SEM Imaging

**Figure 5:**
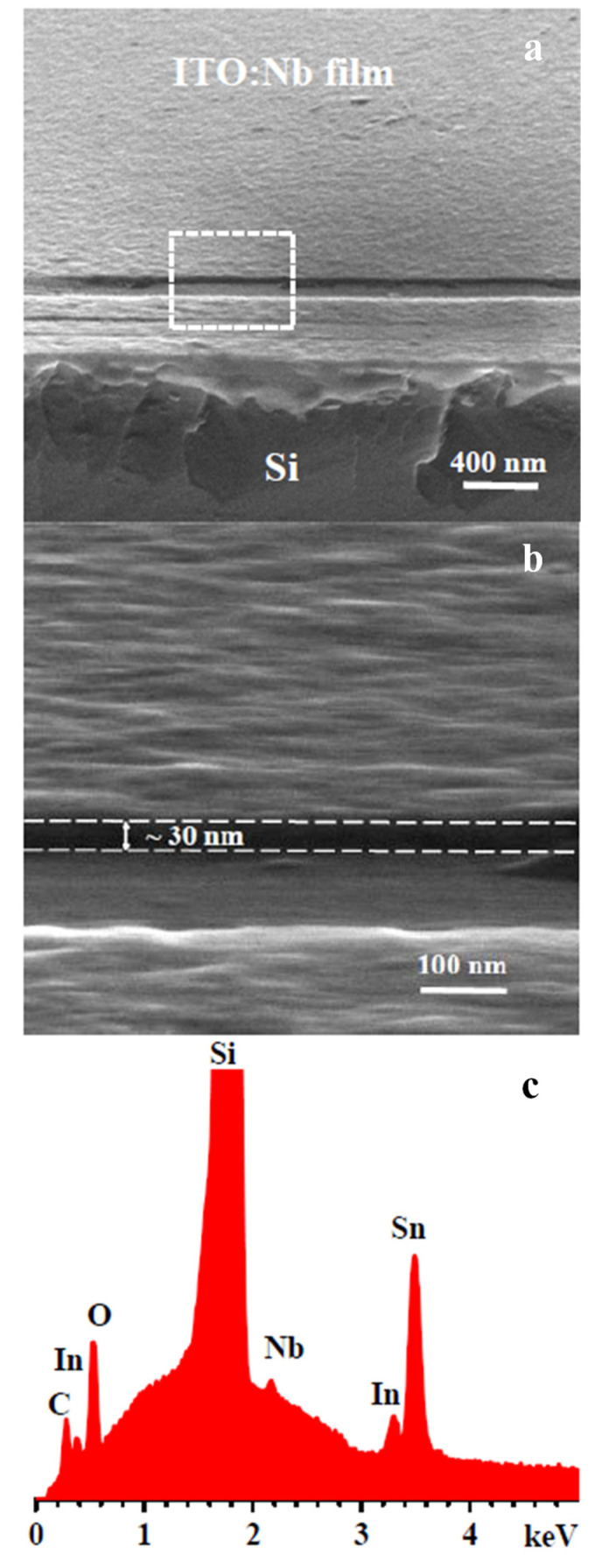
SEM micrographs at different magnifications: ×100,000 (**a**), ×400,000 (**b**), and EDX spectrum (**c**) of ITO:Nb film on Si substrate.

Additional structural measurements of surface texture and film thickness, by SEM and elemental composition, by EDX spectroscopy were carried out on an ITO:Nb film deposited on Si, which was scratched along the edge to expose the film cross-section. Figure 5a shows a tilted view of the film edge at lower magnification (×100,000). It can be seen that the deposited film is continuous, neatly covering the top surface of the Si substrate, and that the surface roughness of the film is in the scale of a few nm, confirming the more precise AFM measurements. The edge of the film along the scratch line has a uniform thickness, about 30 nm, determined in higher magnification (×400,000, Figure 5b). The film surface exhibits a wavy pattern, attributed to the polycrystalline structure of the film, compared with the flat Si surface underneath. Porosity is not observed even at the higher magnification (Figure 5b). The morphology observed in the SEM micrographs is similar to those reported for undoped and Zn-doped ITO films after five layers of deposition on Si substrate [3].

Elemental composition analysis by EDX spectroscopy (Figure 5c) detected the L_α_ bands of In, Sn, and Nb, along with the very intense Si K_α_ peak from the substrate. Due to the very small film thickness (~30 nm), which is at least an order of magnitude thinner than the depth of the zone analyzed by EDX, the Si signal predominates in the EDX spectra. Therefore, the Si K_α_ peak in Figure 5c is truncated. The K_α_ bands of O from the film oxides and C, most probably from atmospheric deposition at the exposed surface, are also detected.

The EDX concentration of constituent elements in atomic percent of the studied sol–gel ITO:Nb(Zn) films [3,22] are given in Section 3.1.

### 2.4. Optical and Photonic Characterization

#### 2.4.1. Raman Spectroscopic Analysis

**Figure 6:**
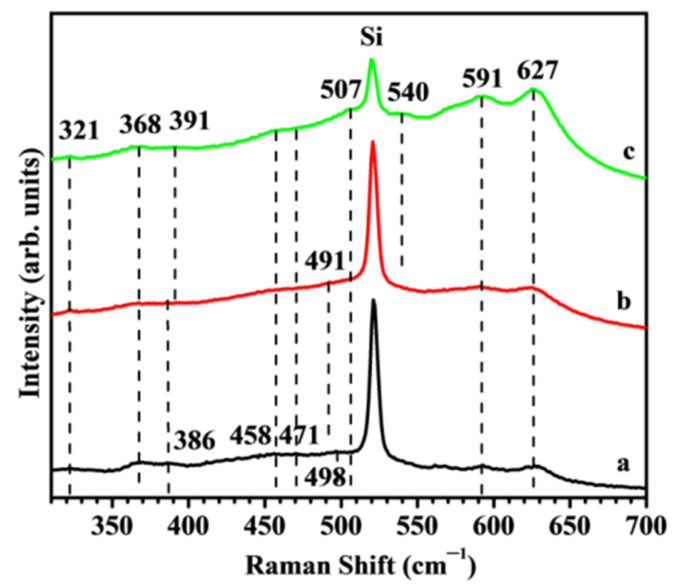
Raman spectra of sol–gel films on Si substrate: ITO (**a**—black), ITO:Nb (**b**—red), and ITO:Zn (**c**—green).

Figure 6 shows the micro-Raman spectra of undoped, Nb-doped, and Zn-doped ITO films. Indium tin oxide and cubic In_2_O_3_ structures belong to the Ia3, Th7 space group. Factor group analysis predicts up to 22 Raman active modes: 4Ag + 4Eg +14Tg. In the Raman spectra (Figure 6) several modes are observed, marked with lines as a guide to the eye. The bands of ITO thin films are much weaker than the intense Si band at ~521 cm^−1^, which can be used as an internal standard for the position of the bands.

The characteristic Raman bands of In_2_O_3_ at 307–308, 365–368, 407, 471, 495–517, and 630–637 cm^−1^, according to the literature [29,30,31,32], can be correlated with the modes observed in Figure 6 at 320, 368, 391, 470, 490–507, and 627 cm^−1^. The bands at 454 and 588 cm^−1^ are attributed to the presence of Sn atoms in the ITO [32]. Raman spectra are in good agreement with modes reported for ITO and ITO:Zn films [3].

The resonant UV Raman spectral features of Zn-doped ITO:Zn films were already discussed in ref. [3]. The observed presence of an additional band at 540 cm^−1^ and, overlapping with the Si band, the mode observed here at 507 cm^−1^, shifted from the undoped ITO mode (498 cm^−1^), which is attributed to the presence of Zn-O bonds.

The Raman spectrum of Nb-doped film shows similar features to the undoped ITO film. The only observed difference is a slight shift in the third mode from 391 to 386 cm^−1^ and the presence of two bands in the 454–470 cm^−1^ range. The characteristic Raman spectrum of Nb_2_O_5_ exhibits a strong feature at 695 cm^−1^ [33], assigned to the symmetric stretching mode of an ordered niobia polyhedral; therefore, the observed Raman results discard the formation of ordered Nb-O6 polyhedra, confirming the absence of crystallized Nb oxide, as indicated by the XRD pattern. Since the ionic radius of Nb^5+^ is smaller than that of In^3+^ ions, the doping of Nb^5+^ in In^3+^ sites could result in lattice deformation [30]; however, Lozano et al. [34] has suggested that Nb^5+^ doping takes place through the formation of Nb_2_O_4_ molecules by drawing extra interstitial O_i_ atoms to gather around the substitutional Nb_In_ sites. Krishnan et al. [35] have reported that moderate doping of Nb enhances the crystalline quality of In_2_O_3_ films. The Micro-Raman analysis reveals the incorporation of Nb^5+^ in the ordered ITO lattice for 4 at.% Nb concentration. The presence of the characteristic bands of cubic bixbyite-type cubic structure with similar spectral features confirms that Nb doping helps to stabilize the ITO-based lattice. Although the size of the Nb^5+^ cation is smaller than the In^3+^ cation, the minimal reduction in the interplanar spacing obtained from XRD analysis comparing the undoped and Nb-doped ITO films can be explained by the presence of interstitial oxygen extracted by the higher valence cation (Nb^5+^) in some Nb_In_ substituted sites. This leads to the increase in the effective size of the Nb-substituted ITO lattice to values close to those of the In^3+^ cation sites.

#### 2.4.2. Spectroscopic Ellipsometric Characterization

The SE analysis of undoped and Zn- and Nb-doped ITO thin films in the 300–1700 nm spectral region deposited on glass, SiO_2_/glass, and Si substrates was used to determine the optical constants (*n, k*), the film thickness (*t_ox_*), the roughness (*t_rough_*), the porosity (*P*), the optical band-gap energy (*E_g_*), the transmittance (*T*), the mobility (*µ_SE_*), and the conductivity (*σ_SE_*), as obtained by modeling the SE data [3,22,27,36]. The experimental Ψ and Δ spectra were simulated with a two-layer model (roughness layer/film/substrate) for samples deposited on glass and a three-layer model (roughness layer/film/SiO_2_/substrate) in the case of films deposited on SiO_2_/glass and Si substrates. The optical properties for the undoped and Zn(Nb) doped ITO films were analyzed based on a General Oscillator model, combining the Tauc-Lorentz (for UV-VIS range) and Drude model (for NIR range). The surface roughness layer was modeled by the Effective Medium Approximation (EMA) [37], consisting of 50% voids and 50% ITO film. The quality of the fit was assessed by the Mean Squared Error (MSE), used to quantify the difference between the experimental and the modeled data. A smaller MSE implies a better model fit of the experimental SE data [37,38].

**Figure 7:**
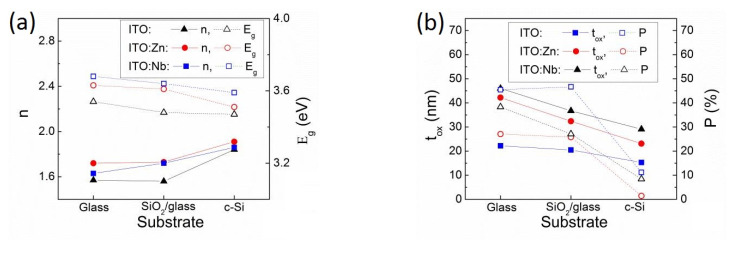
Refractive index (*n*), optical band gap (*E_g_*) (**a**); and film thickness (*t_ox_*), porosity (*P*) (**b**); obtained from the SE data analysis, for undoped and doped ITO thin films on different substrates.

In Figure 7, the variation in the refractive index and the energy band gap are given in relation to the substrate type for the doped and undoped thin films, alongside the thickness and porosity.

The analysis shows a correlated increase in the refractive index with the decrease in band gap energy depending on the dopant and substrate in the order ITO < ITO:Nb < ITO:Zn and glass < SiO_2_/glass < Si, respectively. For the ITO films, the *E_g_* values vary slightly with the type of substrate in the following order: 3.47 eV for Si < 3.48 eV for SiO_2_/glass < 3.54 eV for glass. The differences are within 0.05 eV, which is the accuracy of determining the *E_g_* value. The same tendency is observed for the Nb(Zn)-doped ITO films. However, compared to undoped ITO, the doped films have a larger *E_g_* value. In this case, the difference is beyond the accuracy of the determination method. By doping the films, the number of free charge carriers increases which contributes to the widening of the energy band gap of ITO:Zn(Nb) films (according to the Moss–Burstein effect [39]). These results are also in accordance with the XRD and Raman observations of a higher degree of crystallinity and better structural ordering. The highest values (*E_g_*(Si) = 3.59 eV to *E_g_*(glass) = 3.68 eV) are observed for Nb-doped films, where the Raman spectral analysis (Figure 6) points out that the incorporated Nb atoms contribute to the stabilization of the ITO structure and improve the crystalline quality.

The thickness and porosity of the films as a function of the substrate type are presented in Figure 7b. The smallest film thickness was observed in the case of Si substrate. This can be due to the ordered growth of the films on the highly crystalline Si substrate, leading to a thinner film. As a general observation, doping leads to thicker and less porous thin films. This is confirmed by the reduced film roughness obtained through AFM measurements.

**Figure 8:**
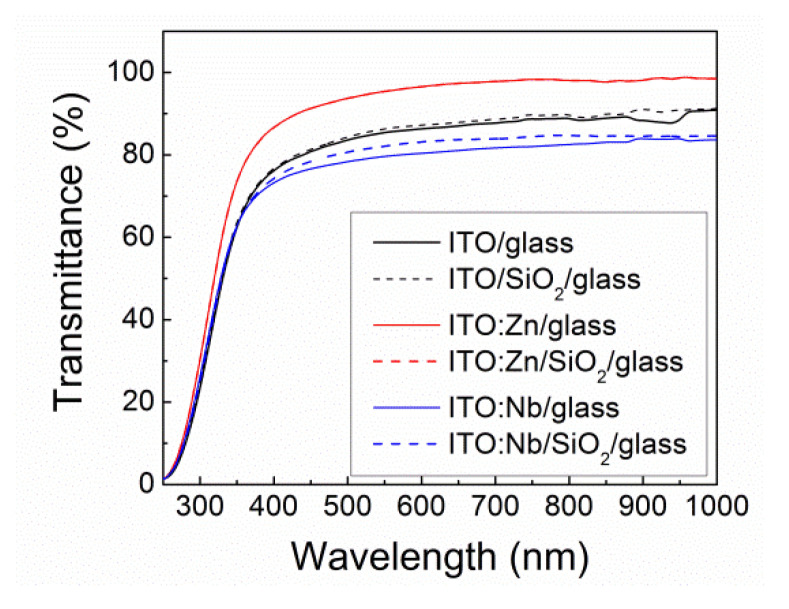
Optical transmittance of undoped and doped ITO films deposited on glass and SiO_2_/glass substrates, respectively.

The optical transmittance (*T*) spectra of the undoped and Zn(Nb) doped ITO films deposited on glass and SiO_2_/glass substrates measured in the 250–900 nm spectral range are shown in Figure 8. All the films exhibit a good transmittance of around 75–95% in the visible range (400–780 nm). Based on the obtained *n* and *E_g_* values, it is expected that the ITO:Nb sample deposited on glass would exhibit the highest transmittance in the VIS range. However, Figure 8 shows that Nb-doped films have the lowest transmittance in the investigated domain. There may be some (limited) surface scattering of the light, due to porosity or surface roughness or, there may be trapping of light at defect states with energetic levels in the ITO bandgap. Generally, the films deposited on SiO_2_/glass substrate are thinner than those deposited on glass, which can be linked to the ordered growth that is more prevalent on SiO_2_/glass. This is directly linked to the transmittance of the films, which is generally higher for the films deposited on SiO_2_/glass substrate.

**Figure 9:**
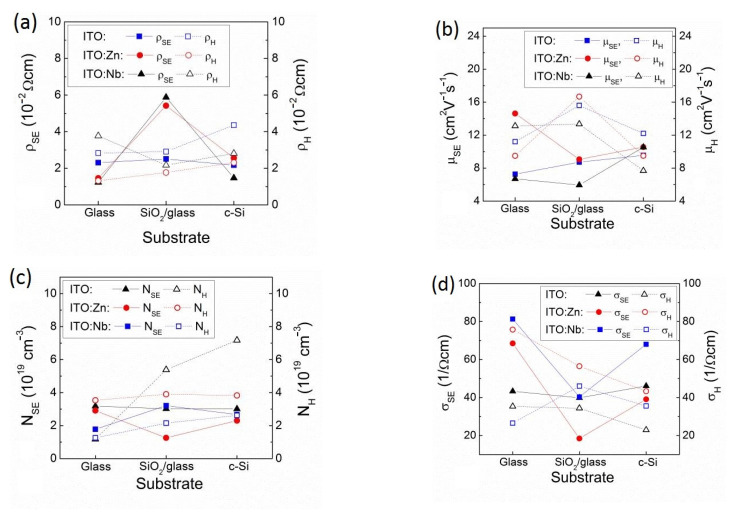
Electrical parameters: specific resistivity (*ρ*) (**a**), mobility (µ) (**b**), carrier concentration (*N*) (**c**), and conductivity (σ) (**d**), obtained by SE analysis for undoped and Nb(Zn) doped ITO films deposited on glass, SiO_2_/glass, and Si substrates; compared with those obtained by Hall measurements from [3,22].

Continuing the SE analysis using the Drude model [38,39], the carrier concentration (*N_SE_*), mobility (*μ_SE_*), resistivity (*ρ_SE_*), and optical conductivity (*σ_SE_*) were obtained (Figure 9), which were compared with the corresponding ones from the Hall effect measurements in our previous studies [3,22].

From Figure 9, it can be seen that the SE and Hall values of the specific resistivity, charge mobility, and carrier concentration are within the same order of magnitude, indicating an appropriate choice of models for SE data processing. Small differences between the results of the two methods may be related to the scattering of charge carriers at the grain boundaries or charge trapping at the defect states, to which the Hall measurement is more sensitive.

The obtained specific resistance values (Figure 9a) show a tendency to increase with the type of substrate in sequence: glass < SiO_2_/glass < Si substrates. The observed discrepancy in the data for the doped samples on SiO_2_/glass can be attributed to many factors, such as surface roughness, structural defects (grain size and degree of crystallinity), chargeable defect states, etc. The SE measurement method is more sensitive to all of these. Nevertheless, the results in Figure 9a correlate well with the observed reduced charge mobility in the corresponding films, which is due to the increased light scattering from the greater surface roughness observed by AFM and the higher porosity determined by SE (Figure 7b). The way in which the dopant is incorporated into the ITO matrix can also influence the electrical properties of the films. XRD analysis shows that Zn is incorporated through substitution, while Nb is incorporated at interstitial sites. This can lead to the limited ability of Nb ions to contribute free electrons and, therefore, explain the overall increased specific resistivity.

### 2.5. Electrical Characterization of Al-ITO-Si Structures

In order to study the electrical active defect states in sol–gel ITO films which affect the charge carrier transport through the films, we measured the electrical characteristics of Al/ITO/Si structures with ITO films deposited on p-Si substrates. Since the thickness of the studied ITO layers is in the 15–30 nm range some undesirable effects, such as Fowler-Nordheim or trap-assisted tunneling of electrons could be expected.

**Figure 10:**
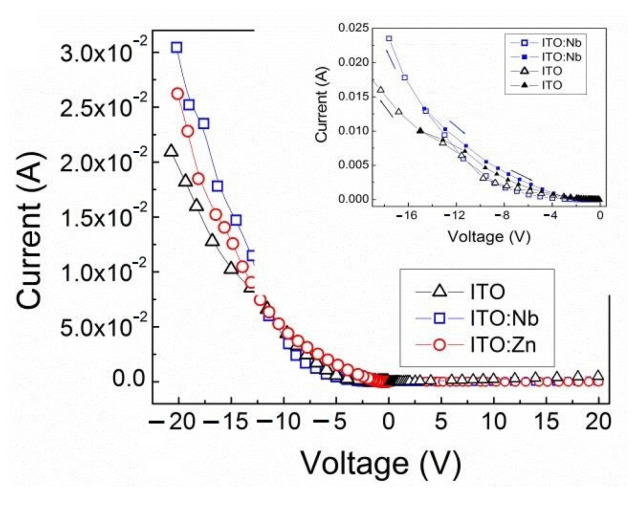
*I-V* characteristics of Al/ITO/Si structures with undoped (triangles) and Nb (squares) and Zn (circles) doped sol–gel ITO films. In the insert, the hysteresis effect is illustrated for the undoped and Nb-doped ITO films.

The measured *I-V* characteristics of the formed Al/ITO/Si structures are given in Figure 10. They show a non-ohmic behavior and are characterized by a well-pronounced asymmetry with respect to the sign of the voltages applied to the upper Al-dot contacts. The current through all ITO films is over two orders of magnitude higher when negative voltages are applied to the upper Al-dot electrodes in comparison with the current when positive voltages are applied to the same dot electrodes. This means that the current of holes from the p-type Si substrate to the ITO film at negative voltages is higher than the minority carrier electron current from the p-type Si substrate to the ITO film at positive voltages. The currents at negative voltages are denoted as forward currents. The current in the undoped ITO film is smaller than the currents of ITO films doped with either Nb or Zn (Figure 10). The reverse currents, measured at positive voltages applied to the Al-dot contacts, practically coincide for the un-doped and Nb(Zn) doped ITO films.

It is also observed that the currents recorded during a forward measurement cycle at upward and backward sweep directions differ from each other, forming a small hysteresis. The hysteresis effect is negligible when the measurement is performed in reverse direction. This is illustrated in the insert of Figure 10 for the Al/ITO/Si structure with undoped and Nb-doped ITO films, with the arrows indicating the sweep directions. The *I-V* dependences for the ITO:Zn film have a similar behavior. As can be seen, the backward current in the hysteresis is higher than the upward one at the same voltage values. This is indisputable evidence for the presence of carrier traps in the sol–gel layers, which are charged during the upward sweep and discharged during the backward sweep. The hysteresis effect is similar for the doped ITO:Nb(Zn) films, while it is smaller for the undoped ITO film.

The concentration of these defect states depends on the degree of crystallinity and porosity of the sol–gel films. The above observations correlate well with the XRD results that revealed nanocrystalline ITO structure with the largest nanocrystallites (10.5 nm) in undoped ITO and decreasing size for doped films followed in sequence: ITO:Zn (9.1 nm) and ITO:Nb on Si (8.4 nm) (see Table 2). The smaller the crystallite sizes, the more grain boundaries there are and, accordingly, the higher the concentration of the electron traps. This may be the reason that, despite Nb(Zn) doping, no significant increase in current density was observed in doped ITO films (Figure 10). For the same reason, the porosity of these films obtained from SE data analysis (Figure 7b) may also contribute to this.

**Figure 11:**
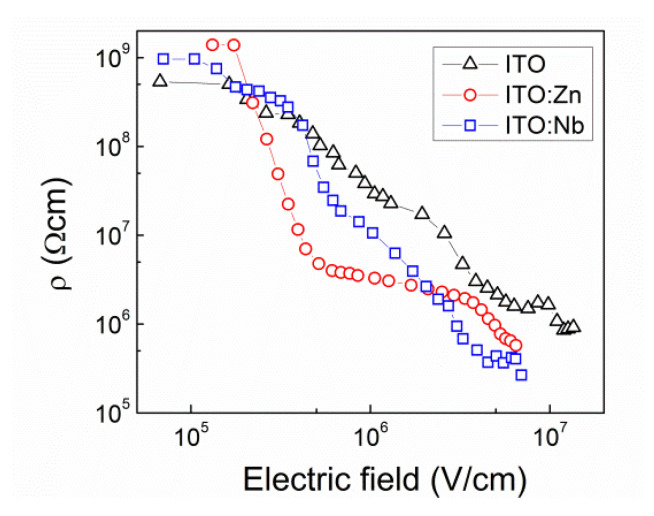
Specific resistivity (*ρ*) versus electric field applied to the Al/ITO/Si structures with undoped ITO (triangles) and Nb (squares) or Zn (circles) doped ITO multilayers.

From the forward *I-V* characteristics given in Figure 10, the differential resistance values (*R = dV*/*dI*) were calculated and the corresponding specific resistivity (*ρ = RA*/*t_ox_*) values were estimated, where *A* is the contact area and *t_ox_* is the thickness of the ITO multilayer. In Figure 11, the *ρ* values are given as a function of the electric field applied to the Al/ITO/Si structures. As can be seen, at low electric films the resistance values are considerably high and they rapidly decrease by nearly four orders of magnitude with the increase in the electric field, but they remain over 10^5^ Ω·cm. The decrease of *ρ* values with increasing applied voltage is clear evidence that the current in these films is limited by the space charge of traps in these films [40,41].

**Figure 12:**
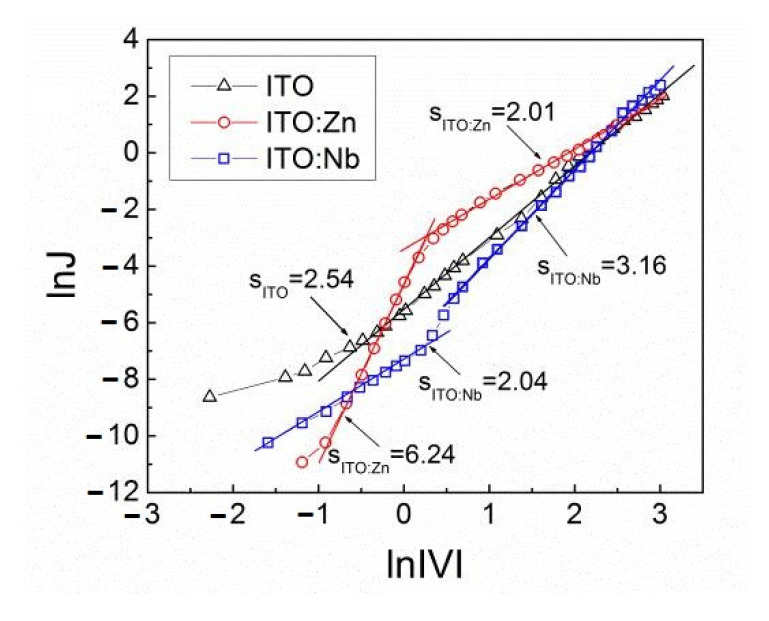
Logarithm of room-temperature forward current density as a function of logarithm absolute voltage for the Al/ITO/Si structures with undoped ITO (triangles) and Nb (squares) or Zn (circles) doped ITO films.

In order to establish the role of traps in the studied ITO films, the forward *I-V* characteristics are plotted in a logarithmic presentation (Figure 12). For the Al/ITO/Si structure with undoped film, the ln*J* versus ln*V* plot has only one predominant slope, the value of which is s = 2.54. For the ITO:Nb and ITO:Zn films, the plots have two dominant parts with slopes higher than 2.

According to the Mott–Gurney law [42,43], when the slop ln*J* versus ln*V* is equal to 2 the current is carried by free charge carriers in the films and it is denoted as space charge limited current (SCLC). When the increase in applied voltage is accompanied by an intensive increase in current, the ln*J* versus ln*V* plot has a much steeper slope than 2 (s > 2). In this case, the current is carried out via traps and it is denoted as trap charge limited current (TCLC) [40,41,42].

In accordance with the above reasoning, both types of current are observed in our ITO layers (Figure 12). In the undoped ITO film (s = 2.54), the current is trap charge limited current (TCLC). In the Nb(Zn) doped ITO films, both the space charge limited (SCLC) and trap charge limited (TCLC) currents are observed, but in different voltage regions. For the ITO:(Nb) at ln/V/ < 0.5, the slope is 2.04, and therefore the current is SCLC. At ln/V/ > 0.5 V, the slope is 3.16 and the current is TCLC. For ITO:(Zn) films at ln/V/ < 0.5 V, the slope is 6.24 and in this case, the current is TCLC, while at ln/V/ > 0.5 V the slope is 2.01 and the current is SCLC.

**Figure 13:**
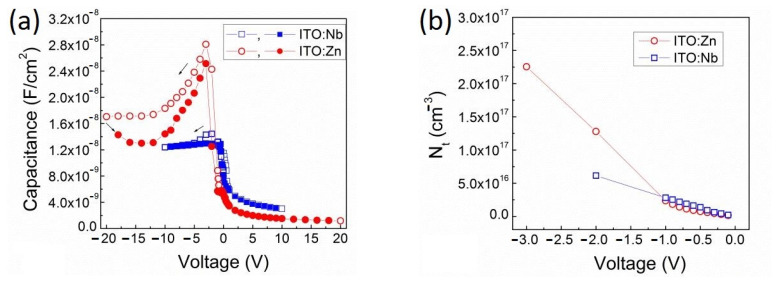
1 MHz *C-V* characteristics of Al/ITO:Nb(Zn)/Si (**a**) and the density of occupied traps (*N_t_*) in ITO:Nb(Zn) as a function of applied voltage (**b**).

In Figure 13a, the *C-V* characteristics of Al/ITO/Si structures with Nb and Zn doped sol–gel films measured at 1 MHz are presented. The curves are quite steep and only slightly stretch toward the negative voltage direction, indicating a good quality of the Si–ITO interface. However, the existence of a well-pronounced broad peak for all ITO films is evidence of a large amount of defect states in the ITO bulk, which are most probably interface traps at the nanocrystalline grain boundaries. The energy levels of these traps are located below the quasi-Fermi level in the ITO energy gap and, at an applied voltage of *V*, they are charged enhancing the capacitance values. The ascending part of the peak starts from about zero voltage up to maximal capacitance (*C_m_*) and its descending part extends up to the capacitance values (*C_ox_*) in the accumulation region.

For the ITO:Zn film, the depletion region is situated close to 0 V and the hole accumulation region is situated between −12 and −20 V (Figure 13a). The broad peak with a maximal capacitance (*C_m_*) is observed at −3 V. The ascending part of this peak is connected with the increased hole capture on the traps present in the ITO film until the electric field reaches 1.298 × 10^6^ V/cm. At this field, the maximal capacitance is equal to *C_m_* = 2.81 × 10^−8^ F/cm^2^.

For the ITO:Nb film, the depletion region is also situated close to 0 V but the hole accumulation region is started already from −6 V (Figure 13a). The observed peak is rather broad but smaller, and the maximal capacitance of *C_m_* = 1.46 × 10^−8^ F/cm^2^ appears at −2 V when the field reaches 6.87 × 10^5^ V/cm.

The voltage dependence of the density of the occupied traps (*N_t_*) on the ascending side of the peaks is presented in Figure 13b. These values are determined using Equation (1):

*N_t_*(*V*) = *C*(*V*) × *V*/(*qAt_ox_*) (1)

In the ITO:Zn film, the calculated maximal density (*N_tm_*) value of the occupied traps is equal to *N_tm_* = 2.25 × 10^17^ cm^−3^, while in the ITO:Nb film the *N_tm_* value is equal to 6.13 × 10^16^ cm^−3^.

Figure 13b shows that the concentration of captured charges is linearly proportional to the applied electric field. In ITO:Nb, the *N_t_* versus *V* dependence is linear up to reaching the *C_m_* at −2 V, while for the ITO:Zn there are two slopes. At small voltages, the slope is similar to that obtained for ITO:Nb but over −1 V the slope becomes much steeper. At a higher electric field than for *C_m_*, the concentration of trapped charges decreases, alongside the decrease in the capacitance in the descending part of the peak (Figure 13a). Such linear dependences prove that the calculation of the concentration of occupied traps by Equation (1) is valid, not only for the maximal capacitance *C_m_* but also for the entire range of the applied voltage.

The obtained *N_t_* values are considerably smaller than the density of free charge carriers determined by SE and Hall measurements (Figure 9c). This discrepancy is related to the different transport mechanisms for trapped and free charges.

**Figure 14:**
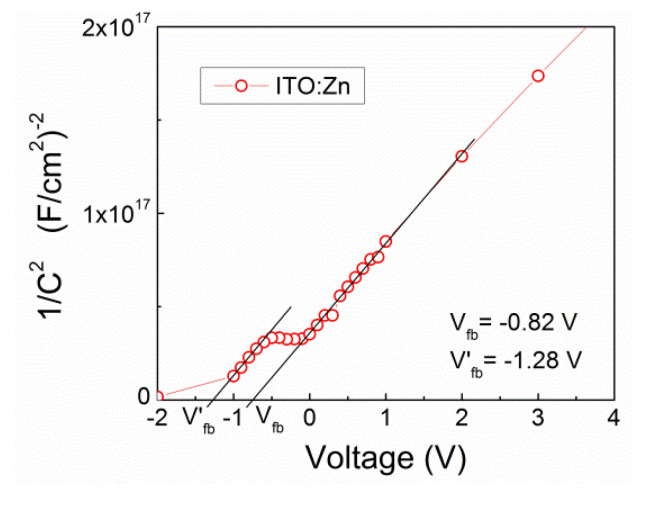
Mott–Schottky plot represented by Al/ITO:Zn/Si structure.

The Mott–Schottky plot [44] represented by the reciprocal of the squared capacitance as a function of applied voltage for the Al/ITO:Zn/Si structure is given in Figure 14. In the depletion region, the 1/C^2^ versus V plot is linear and from its slope, one can determine the doping density of the Si substrate if it is unknown. The intersection with the voltage axis yields the flat-band voltage build-in by fixed charges in the defect states at the Si–ITO interface. As is seen in Figure 14, at the Si–ITO:Zn interface, the discharging of defect states creates a plateau in the 1/C^2^ versus V plot that shifts the flat-band potential position toward larger negative voltages from *V_fb_* = −0.82 V to *V’_fb_* = −1.28 V, indicating that this kind of defect states generated by Zn dopants are positively charged.

Analogously to the above procedure, from the 1/C^2^ versus V plot for the Al/ITO:Nb/Si structure, the *V_fb_* and *V’_fb_* values were determined. In comparison to ITO:Zn film, the flat-band potential position for the ITO:Nb shifts to the opposite direction (from *V_fb_* = −1.62 V to *V’_fb_* = −1.17 V), proving that the introduction of Nb dopant into ITO creates negative charges at the Si–ITO:Nb interface. The different signs of the built-in charges at the Si–ITO:Nb(Zn) interfaces testify to different origins of these electrically active defects being dependent on the type of the dopant atoms.

The densities of fixed charges (*N_f_*) and interface traps (*N_it_*) are calculated using the formulas in Equation (2).

*N_f_* = (*V_fb_* − *V^i^_fb_*) × *C_ox_/q; N_it_* = (*V* − *V^i^_fb_*) × *C*(*V*)/*q* (2)

where *V^i^_fb_* is the ideal flat-band voltage (zero defect states at the interface), and summarized in Table 3. Knowing the Si substrate doping (*N*_a_ = 1.5 × 10^15^ cm^−3^) and the *E_g_* value the ideal flat-band voltage can be obtained by

*qV^i^_fb_* = *φ_m_* − *χ* − *E_g_* + *kTln*[*N_v_*(*T*)/*N_a_*] (3)

where *φ_m_* is the metal work function, *χ* is the electron affinity, and *N*_v_ is the effective density of states in the Si valence band. At the Si–ITO:Zn interface, the fixed positive charge density is considerably low and equal to *N_f_* = 8.92 × 10^10^ cm^−2^, while at the Si–ITO:Nb interface the fixed negative charge density is equal to 8.05 × 10^10^ cm^−2^. The interface trap densities (shallow surface states) at the Si–ITO interface are in the order of 10^10^ cm^−2^ for both kinds of Al/ITO/Si structures.

The observed defect states at the Si–ITO interface most probably originated from trivalent Si dangling bonds (Si≡Si•), being a source of electron traps, and unsaturated Si bonds in O_3_ = Si•, being a source of fixed positive charges, which are dominant defect states in the Si surface region [45].

**Figure 15:**
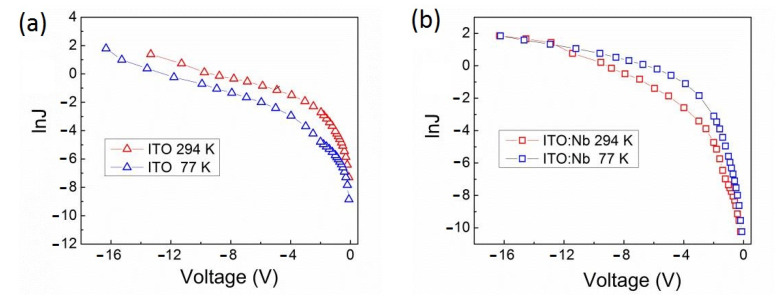
*I-V* characteristics of Al/ITO/Si structures with undoped ITO (**a**) and Nb-doped ITO (**b**) films, measured at temperatures of 294 K and 77 K.

The *I-V* characteristics of the studied Al/ITO/Si structures were also measured at 77 K. In Figure 15, the *I-V* curves are represented by the undoped ITO (Figure 15a) and Nb-doped ITO (Figure 15b) films, where they are given as logarithm of the forward current density versus applied voltage. For the Al/ITO:Zn/Si structure, the ln*J* versus *V* curves are similar to those of Al/ITO:Nb/Si and are not given.

There are small differences between the corresponding characteristics measured at room temperature (294 K) and at 77 K. Such temperature dependence points out that the current implements in a wide low-temperature range by variable-range hopping of charges from occupied to non-occupied states localized close to the Fermi level in the ITO bandgap [46,47]. As is seen from Figure 15b, in the voltage range −1 V and −11 V, the low-temperature (77 K) current densities in the doped ITO films become slightly higher than those measured at room temperature. This increase in the current density at 77 K can be related to the enhanced value of *N_t_* at the position of quasi-Fermi level in the ITO bandgap moving from its room temperature position by temperature.

The effective activation energy (*qφ_a_*) of the current densities is estimated from the expression ln[*J*(*T*_1_)/*J*(*T*_2_)] = (*qφ_a_/k*) × [(1/*T*_2_) − (1/*T*_1_)] and is summarized in Table 3. All the obtained energy values are much lower than the thermal energy value of kT~25 meV, indicating that the current does not occur by thermally activated carrier hopping. For the undoped ITO film, at a low electric field of 2.62 × 10^5^ V/cm (−0.4 V), the activation energy is equal to 13.5 meV and decreases to 2.3 meV at a high electric field of 6.54 × 10^6^ V/cm (−10 V). For the Nb(Zn) doped ITO films, the *qφ_a_* values are between 4.6 and 7.7 meV.

## 3. Materials and Methods

### 3.1. Film Preparation

The undoped and Nb- or Zn-doped ITO films (ITO:Nb_2_O_5_ and ITO:ZnO) were prepared by the dip-coating sol–gel method using a solution with 0.1 M concentration. The following chemicals were used as precursors: indium nitrate as In_2_O_3_ source, 2 tin ethyl hexanoate as SnO_2_ source, niobium (V) ethoxide or zinc nitrate as dopants, 2,4-pentanedione as chelating agent, and ethanol as solvent. All the precursors were purchased from Merck (Darmstadt, Germany) with 99.99% purity for each.

**Figure 16:**
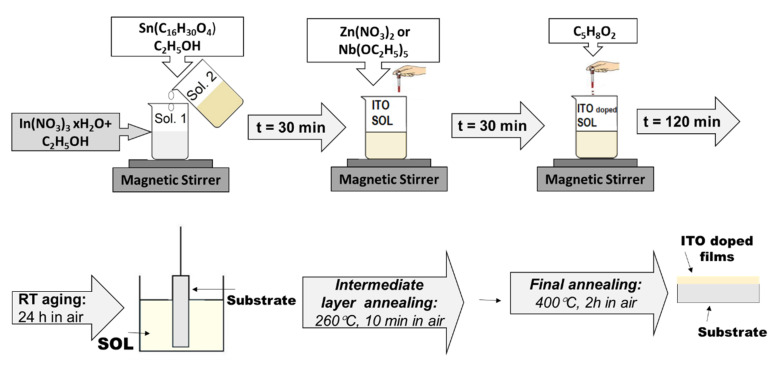
The flowchart of sol–gel technological procedures for the preparation of undoped and doped ITO thin films.

The technological procedures are schematically illustrated in Figure 16. The chemical reactions proceeded at room temperature under stirring, with the duration of each given in Figure 16. The prepared solutions were aged for 24 h in the air at atmospheric conditions before film deposition.

In the present study, the ITO films were deposited on microscopic glass, SiO_2_/glass, and Si(100) substrates. In the case of the SiO_2_/glass substrate, the glass was coated with SiO_2_ by the sol–gel technology described in our previous work [33]. In this way, we intended to prevent the possible diffusion of impurity elements from the glass substrate into the ITO layers. The Si substrates were p-type, (100) oriented wafers with specific resistivity of 8–10 Ω·cm corresponding to ~1.5 × 10^15^ cm^−3^ impurity concentration.

The multilayered ITO films were obtained by five successive deposition cycles using the dip-coating method with a withdrawal rate of 5 cm/min. After each intermediate layer deposition, it was annealed at 260 °C for 10 min in air. After the deposition of the last (5th) layer, the structure was stabilized at 400 °C in air for 2 h.

The elemental composition of the prepared sol–gel films has been determined by EDX measurements given in [3,22]. Briefly, for undoped ITO films, the In:Sn ratio was found ~ 5:1. In the ITO:Zn film the concentration of Zn was 4 at.% [3] while, in ITO:Nb, the Nb content was 3.7 at.% [22].

### 3.2. Film Characterization

The structure of undoped ITO and ITO:Nb and ITO:Zn films was studied by analysis of the X-ray diffraction (XRD) patterns recorded on a Rigaku Ultima IV equipment (Rigaku Corp., Tokyo, Japan) operated with Cu Kα radiation (λ = 0.15405 nm). Due to the nanoscale thickness of the films, the measurements were performed using thin film geometry, at a fixed incidence angle of ω = 0.5°, with a scanning rate of 5°/min over a range of 20–70°. Crystallite sizes were obtained using Scherrer’s formula and the diffraction line assignments were given by the search/match algorithm connected to the ICDD database.

The surface morphology of the sol–gel films was studied by Atomic Force Microscopy (AFM) measurements, carried out in the non-contact mode using sharp tips on an XE-100 apparatus from Park Systems Japan Inc. (Japan, Tokyo). The topographical 2D AFM images were taken over a scanned area of 1 × 1 µm^2^. The images were processed with the XEI (v.1.8.0) Image Processing Program developed by Park Systems regarding the statistical analysis of the AFM micrographs.

SEM micrographs were taken on an SEM FEI Quanta 3D microscope (Eindhoven, The Netherlands) operating in the range of 5 and 20 kV equipped with a dispersive energy X-ray spectrometer (EDX).

Raman vibrational spectroscopy measurements of undoped ITO and doped ITO:Nb and ITO:Zn films deposited on silicon were carried out in a LabRAM (Horiba Jobin Yvon, Tokyo, Japan) in the 300 and 700 cm^−1^ Raman Shift range, using the 325 nm emission of a He/Cd laser as excitation line (NUV 40X objective, spot size ~1–2 μm).

Spectroscopic ellipsometry (SE) measurements were performed in UV-VIS-NIR spectral range with J. A. Woollam Co. Inc. (Lincoln, NE, USA) equipment. The SE spectra were measured at an incidence angle of 70° in the (300–1700) nm spectral range with a step of 10 nm. WASE program from Woollam was used for multi-parameter fitting, while for minimizing the difference (mean square error–MSE) between the experimental and theoretical data the iterative least-squares method was applied. From the SE data analysis, the films’ thickness (*t_ox_*) and dielectric constants (refractive index (*n*) and extinction coefficient (*k*)) are determined with an accuracy of ±0.2 nm and ±0.005, respectively. Knowing these parameters and using the Drude model [35], the concentration of free carriers, *N_SE_,* their mobility, *μ_SE_*, and optical conductivity, *σ_SE_,* were determined. The optical band gap energy (*E_g_*) and the porosity (*P*) of the films were calculated using Tauc’s method, by plotting (*αhν*)^1/2^ versus photon energy (*hν*) for indirect transitions [48] as well as using the formula [49] *P* = [1 − (*n*^2^ − 1)/(*n_d_*^2^ − 1)], where *n_d_* = 1.92 is the refractive index of pore-free ITO at λ = 500 nm and *n* is the refractive index of undoped or Zn(Nb) doped ITO films at the same wavelength.

For the electrical characterization of the sol–gel ITO:Nb(Zn) films, metal contacts were formed by vacuum thermal evaporation of Al-dots on the upper surface of the ITO layers and, as backside contact a continuous Al film was deposited on the Si-substrate backside surface. The current-voltage (*I-V*) and capacitance-voltage (*C-V*) characteristics of thus formed Al-ITO-Si-Al structures were measured manually with a measurement cycle duration of approximately 20 min. The measurement sequence started from 0 V toward negative or positive voltages applied to the Al-dot contact on the ITO film surface, followed by a voltage reversal toward zero applied bias voltage. The *C-V* curves were recorded at 1 MHz test signal frequency by a digital LCR meter E7-12. All electrical measurements were performed at room temperature unless otherwise stated.

## 4. Conclusions

Using sol–gel layer-by-layer technique, thin multilayered undoped and Nb(Zn) doped ITO films have been prepared by subsequent deposition of five layers on glass, SiO_2_/glass, and Si substrates. XRD, AFM, SE, and electrical studies were employed to provide a thorough characterization of the obtained films, with insights into their crystallinity, morphology, thickness, and optical and electrical properties.

XRD analysis has revealed the polycrystalline bixbyite In₂O₃ structure of the sol–gel films, supported by the Raman spectral analysis. Doping promotes crystallization, with doped films showing slightly smaller crystallite sizes, possibly due to the larger amount of nucleation centers introduced by the dopants.

AFM imaging has demonstrated the decreasing influence of the substrate on the topography of the deposited ITO films in the sequence from glass, SiO_2_/Si to Si, and from undoped to Zn- and Nb-doped ITO films. The amorphous structure of the glass substrates facilitates the growth of thicker layers but gives the largest RMS values for the deposited ITO films.

SE studies show that the refractive index increases while the band gap decreases in relation to dopant type and substrate, following the order ITO < ITO:Nb < ITO:Zn and glass < SiO_2_/glass < Si, respectively. ITO:Zn films on glass substrate achieve the highest transmittance (90%), while resistivity is highest for the films on SiO₂/glass substrates, possibly due to the lower charge mobility and low charge carrier number, affecting the effectiveness of the chosen SE model. Nb-doped ITO films exhibit the lowest mobility, which could be due to Nb occupying interstitial sites rather than substitutional sites.

The *I-V* characteristics of the Al/ITO/Si structures show well-defined asymmetry with respect to the sign of the applied voltages. The presence of hysteresis in the *I-V* curves is evidence of the existence of carrier traps in the sol–gel films. The density of occupied charge in these traps is in the range of 6 × 10^16^–2.5 × 10^17^ cm^−3^, which correlates with the degree of crystallinity and porosity of the sol–gel films observed by XRD and SE, respectively. However, these densities are significantly smaller than the free charge carriers densities determined from SE analysis. This discrepancy is related to the difference in transport mechanisms for trapped and free-charge carriers.

The current density in the forward direction obeys the relation *J~V^s^*, s being larger than 2. In the undoped ITO film *s* is 2.54 and the current is trap charge limited current (TCLC), while in the Nb(Zn) doped ITO films, the space charge limited (SCLC) (*s*~2) and traps charge limited (TCLC) currents (6.24 > *s* > 3.16) are observed, but in different voltage regions. The weak temperature dependence of the *I-V* characteristics indicates that at low temperatures the charge carrier transport is via variable-range hopping of charges from occupied to unoccupied states localized near the Fermi level in the ITO bandgap.

## Figures and Tables

**Table 1:** Recent developments in ITO-doped and undoped films in the last five years.

Year	Materials	Deposition Method	Applications	Main Results	Refs.
2019	ITO + Fe	Sputtering	TCO	The transmittance of the ITO thin films has significantly improved through Fe doping. Iron dopant leads to a smooth surface compared to undoped ITO.	[18]
2020	ITO + Ga	SG	(TFTs)	The stability under stress tests of Ga-doped ITO TFTs has been improved by using a small amount of Ga as a dopant, compared with the undoped ITO thin films (ITO TFTs).	[19]
2020	ITO + Ce	Sputtering	TCO	The transmittance and electrical conductivity of the ITO thin films doped with 0.5 wt% Ce are enhanced compared to the undoped ITO films.	[20]
2021	ITO + Zr	Co-sputtering	TCO	The transmittance of the Zr-doped ITO thin films was enhanced in the infrared region, while in the VIS * region no important changes occurred, in comparison with the pure ITO films.	[21]
2021	ITO + Mo	RF *	TCO	The effect of thermal annealing on the properties of Mo-doped ITO films on film properties was investigated, observing an improvement of the optical transparency in VIS * and NIR * regions at T ≥ 300 °C.	[2]
2022	ITO + Zn	SG *	CO_2_ sensor	In terms of gas sensing properties, Zn-doped ITO thin films exhibit better sensitivity compared to the undoped samples.	[3]
2022	ITO + Nb	SG *	CO sensor	The Nb doping of ITO thin films was studied in terms of gas sensing. The measurements of undoped and Nb-doped ITO thin films show that the addition of Nb improved the gas detection, with the best results for 2000 ppm CO concentration at a working temperature of 300 °C.	[22]
2023	ITO + Mn	RF * magnetron sputtering		The Mn-doped ITO thin films deposited on single crystal yttria-stabilized zirconia (YSZ) substrates with different crystal plane orientations: (111), (110), (100) display surface uniformity as a result of epitaxial growth. In contrast, the films deposited on the YSZ (111) substrate present interesting properties (crystallinity, surface smoothness, and electrical conductivity).	[23]
2023	ITO + Nb	Co-sputtering	TCO	The addition of Nb as a dopant to ITO thin films increased the optical transmittance in the NIR * region compared to undoped ITO film.	[5]
2022	ITO	SG *	TCO	The ITO thin films were annealed in a reducing gas with the aim of investigating the effect of optical-electrical properties. By such an annealing the electrical performance of the films was improved. Additionally, the annealed films exhibited higher transmission in the range of visible light.	[24]
2023	ITO	PLD	Semiconductor thin-film thermocouples (TFTCs)	The ITO thin films display a nano cone structure and thermoelectric properties, which make them a good candidate for the preparation of thermocouples.	[25]
2024	ITO	RF * magnetron sputtering	Flexible transparent electronic devices	The optical and electrical properties of ITO thin films were investigated at various annealing temperatures and under mechanical bending conditions. It was found that the films exhibit high transparency and good conductivity.	[26]

* RF—RF magnetron sputtering; * SG—Sol–gel; * TFTs—Thin film transistors; * VIS—visible region; * NIR—Near-infrared region; * PLD—Pulsed laser deposition.

**Table 2:** The interplanar spacing (d), full width at half maximum (FWHM), and the crystallite size for the crystal plane with Miller indices (222).

Substrate	Sample	d-Spacing (Å)	FWHM (°)	Crystallite Size (nm)
SiO_2_/Glass	ITO	2.905	0.79	10.9
	ITO:Zn	2.897	0.94	9.1
	ITO:Nb	2.902	0.90	9.6
Glass	ITO	2.907	0.93	9.3
	ITO:Zn	2.896	1.00	8.6
	ITO:Nb	2.898	0.93	9.3
Si(100)	ITO	2.904	0.82	10.5
	ITO:Zn	2.897	0.94	9.1
	ITO:Nb	2.905	1.03	8.4

**Table 3:** Density of electrically active defect states at the Si–ITO interface and in the ITO bulk.

Structure	At Si–ITO Interface		In ITO Bulk		
	*N_f_* (cm^−2^)	*N_it_* (cm^−2^)	*N_tm_* (cm^−3^)	*qφ_a_* (at −0.4 V) (meV)	*qφ_a_* (at −10 V) (meV)
Si–ITO	+6.35 × 10^10^	1.20 × 10^10^	-	13.5	2.3
Si–ITO:Zn	+8.92 × 10^10^	1.37 × 10^10^	2.25 × 10^17^	7.02	6.11
Si–ITO:Nb	−8.05 × 10^10^	2.15 × 10^10^	6.13 × 10^16^	7.68	4.6

## Data Availability

The data presented in this study are available on request from the corresponding authors.
